# Self-assembled nanoparticles: A new platform for revolutionizing therapeutic cancer vaccines

**DOI:** 10.3389/fimmu.2023.1125253

**Published:** 2023-02-21

**Authors:** Tianyu Shi, Mengna Sun, Changchang Lu, Fanyan Meng

**Affiliations:** ^1^ The Comprehensive Cancer Centre of Nanjing Drum Tower Hospital, Clinical College of Traditional Chinese and Western Medicine, Nanjing University of Chinese Medicine, Nanjing, China; ^2^ The Comprehensive Cancer Centre of Nanjing Drum Tower Hospital, The Affiliated Hospital of Nanjing University Medical School, Nanjing, China

**Keywords:** cancer vaccines, nanoparticles, adjuvant, self-assembled, immunotherapies

## Abstract

Cancer vaccines have had some success in the past decade. Based on in-depth analysis of tumor antigen genomics, many therapeutic vaccines have already entered clinical trials for multiple cancers, including melanoma, lung cancer, and head and neck squamous cell carcinoma, which have demonstrated impressive tumor immunogenicity and antitumor activity. Recently, vaccines based on self-assembled nanoparticles are being actively developed as cancer treatment, and their feasibility has been confirmed in both mice and humans. In this review, we summarize recent therapeutic cancer vaccines based on self-assembled nanoparticles. We describe the basic ingredients for self-assembled nanoparticles, and how they enhance vaccine immunogenicity. We also discuss the novel design method for self-assembled nanoparticles that pose as a promising delivery platform for cancer vaccines, and the potential in combination with multiple therapeutic approaches.

## 1 Introduction

Cancer vaccines, typically given through exogenous administration of selected tumor antigens combined with adjuvants that activate dendritic cells (DCs), ultimately aim to stimulate the immune system against tumor antigens. In recent years, the success of therapeutic cancer vaccines has dramatically revolutionized cancer treatments and has provided more alternative approaches to patients with cancer (1, 2). Several clinical trials have also confirmed the potential of this strategy, including those for melanoma, lung cancer, and prostate cancer (3–5). With the discovery of neoantigen target being continually advanced to improve the identification of immunogenic neoepitopes that can be recognized by CD8+ T cells, personalized therapeutic cancer vaccines have been considered in clinical investigation (6). The potential of therapeutic cancer vaccines in tumor immunotherapy has led to growing interest in developing more effective vaccine strategies to achieve tumor regression. However, recent years have seen excellent advances in self-assembled nanoparticle (SANP) platforms for therapeutic cancer vaccines. SANPs can evoke the epitope-specific B/T-cell immune responses through epitope folding similar to microorganisms that can protect payloads from enzymatic degradation, deliver to lymphoid organs, and target the tumor (7, 8). Additionally, antigen loading is also increased with the improved performance of SANPs. Because of the advantages from SANPs, they have naturally become the focus of current vaccine research and have appeared in various medical fields.

As early as the 1950s, rod-shaped particles have been found in tobacco mosaic virus (TMV); this is one of the earliest discoveries in history about self-assembled particles (9). In 1981, Drexler proposed the molecular engineering of proteins, being the monomeric building blocks, through self-assembly (10). Since then, materials based on self-assembled peptides such as nanofibers have been produced (11). Indeed, with the development of nanoparticle (NP) technology, including poly(lactic-coglycolic acid) (PLGA) (12), polymersomes (13), and liposomes (14), the design and manufacture of self-assembled nanovaccines are an empirical process. SANPs are composed of multi-component homologous NPs through non-covalent bonds or weak covalent bond interactions aimed at achieving a stable and balanced state (15). The assembly of different or similar molecules driven by intermolecular forces, including van der Waals interactions, electrostatic interactions, hydrogen bonding, coordination, hydration forces, and solvation interactions, occurs if there is equilibrium between them (11, 16). While these forces are indeed weak when considered individually, mixtures of different interaction types can result in structural and chemical stabilization, and these conformations formed by the free assembly of molecules are easy to accept. Of course, the modified module can also be selected as the substrate, which can control the direction of self-assembly to a certain extent and can directly act between the molecules or the module by external stimuli, including pH, temperature, solvent polarity, electromagnetic radiation, and light, to guide self-assembly (17). NPs designed through synthetic self-assembled technology (SANPs) can be given certain physiological functions. They can be used as platforms to display the arrangement of specific immunogens and orderly matrices to mimic the folding and complex structure of natural microbial surfaces (18). Therefore, SANPs can offer potential advantages such as improved stability due to the co-delivery of an antigen with adjuvant, and constant release and/or specific activation of the immune system (19). To a certain extent, we believe that the limitations of clinical application caused by the defects of the conventional vaccine platforms can be alleviated by SANPs.

The development of SANPs has led to many vaccine platforms and drug delivery systems (17), including broad platforms such as peptides/polymers (20). Generally, antigen loading is chemically defined, and SANPs based on peptide antigens are linked to hydrophobic carriers like peptide, lipids, and polymers. Of course, we also introduce a new way of antigen loading here. In this review, we discuss the characteristics of the internal structure of vaccines based on various self-assembled NPs, such as polymers and lipids for inducing anticancer T-cell immunity, and some SANP-based delivery platforms. Additionally, we also discuss current challenges of therapeutic cancer vaccines and their potential applications when combined with other treatments for overcoming tumor resistance and promoting clinical efficacy.

## 2 The design of SANPs

**Figure 1:**
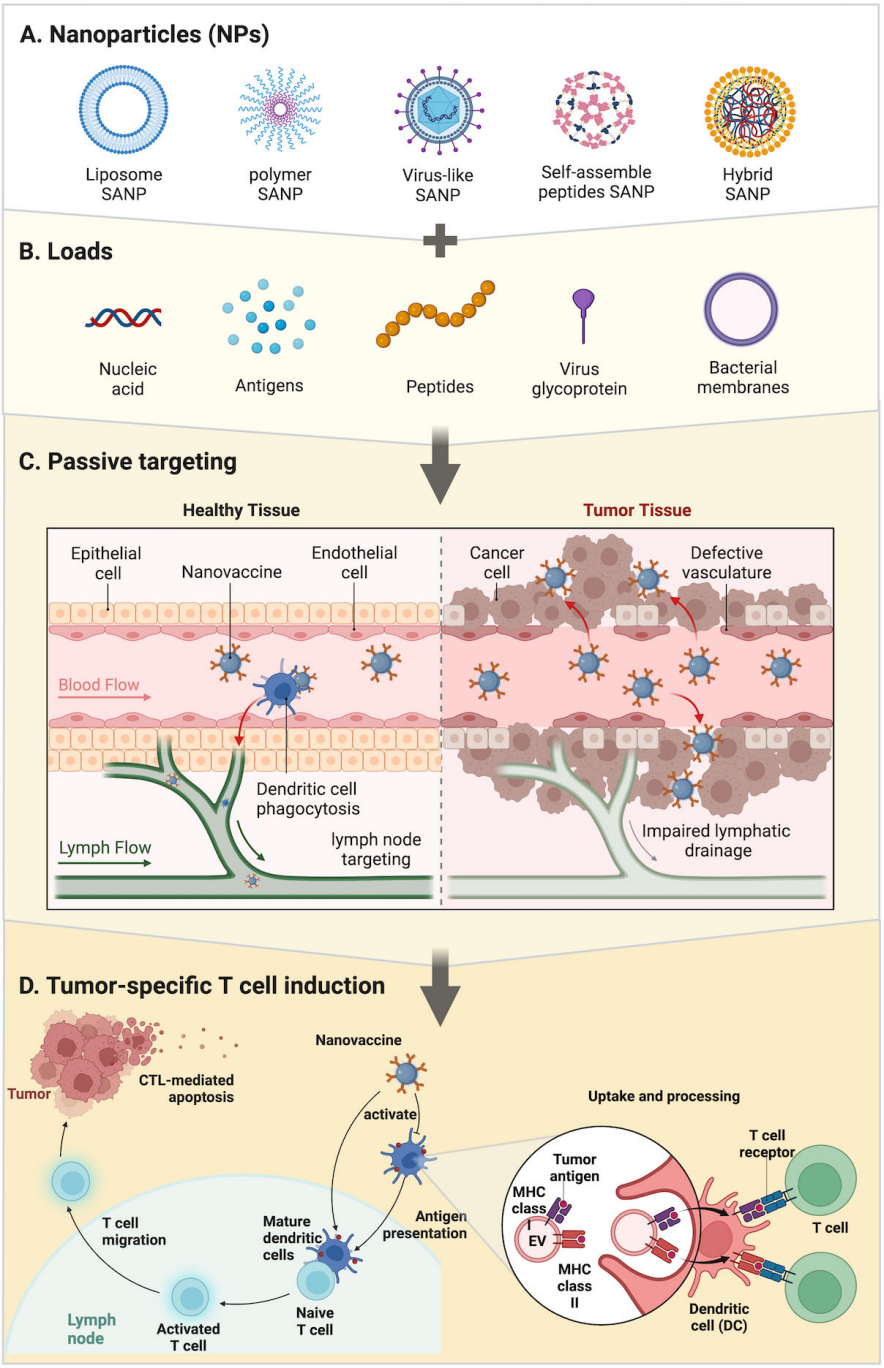
Strategies of SANPs: **(A)** SANPs based on multiple materials. **(B)** During the synthesis of SANPs, antigens or other immune activators can be loaded onto SANPs by chemical modification or physical packaging. Nucleic acids and antigens can be physically embedded in liposome and polymer SANPs; bacterial membrane can wrap around SANPs’ surface; virus glycoprotein and peptides are loaded into viral particles or peptide chains through autonomous synthesis, as well as antigens. **(C)** SANP-based nanovaccines targeting SANPs were injected intravenously into the bloodstream and circulate throughout the body to activate dendritic cells and recruit T cells around lymph nodes and tumor tissue. **(D)** Tumor-specific T-cell induction: SANPs can induce cytotoxic T lymphocytes by activating peripheral dendritic cells (DCs) that then present antigens to T cells in lymph nodes, or SANPs can directly target lymph nodes for induction within lymph nodes. Nanovaccines deliver antigens and adjuvants to DCs, which subsequently upregulate co-stimulatory molecules and present the processed antigen to T cells *via* MHC-II molecules. DCs can directly activate T cells by MHC-bound antigens and costimulatory molecules present on cell-derived membranes on the surface of SANPs.

There are two ways to achieve nanotechnology: top-down and bottom-up (21). The top-down method dissociates NPs from larger structures while maintaining their original composition and properties; the bottom-up method uses assembly or self-assembly to design NPs at the basic molecular level. To design an appropriate antigen carrier that delivers the screened pathogen epitopes and can induce a corresponding response in animal immunity studies model (22, 23). For example, in the PGS-co-PEG NPs system, Ankur and his colleagues synthesized the PGS-co-PEG polymer through a two- step polycondensation reaction (24). The first step, polycondensation reaction of PEG and sebacic acid under stirring condition. In the second step, glycerol was added to obtain a block copolymer of PGSco-PEG (pre-polymer) with different ratio of PEG segments.. The size of NPs can be controlled by controlling the molar ratio of PEG, thereby synthesizing precise nanostructures. To a certain extent, the size of NPs determines the binding and activation of their membrane proteins and receptors (25). Different immune cells have their corresponding SANPs from different dimensions. SANPs of size > 100 nm are mostly absorbed by local antigen-presenting cells (APCs) at the site of injection, as well as tend to accumulate in highly permeable tumor tissue, when employed in prophylactic and therapeutic immunotherapy (26). In contrast, smaller nanostructures (40–50 nm) tend to enter lymphatic vessels and reach lymph nodes (LNs); they can be internalized with greater efficiency by LN-resident DCs, triggering an effective and long-lasting immune response against tumor (26, 27) (**Figure 1C**). The peptide self-assembly process and the size of its production are strongly influenced by amino acid sequences and physicochemical indicators, such as pH (from acidic to slightly alkaline), temperature (4–95°C), metal ion concentrations (such as calcium, potassium, magnesium and sodium), and salt concentrations (28). SANPs can become structurally stable if the environmental parameters are obtained (26). The self-assembled form can be achieved by adjusting the concentration of metal ions. Changing the MgCl_2_ concentration, for example, could encourage carbonic anhydrase (BCA) linked with P114 peptide (BCA-P114) NPs to self-assemble (26). NPs can be obtained by using MgCl_2_ and Tris-HCl as low as 5 mM and 10 mM at neutral pH. The maximum size of these NPs can be formed when MgCl_2_ concentration is 25 mM. When the concentration of MgCl_2_ exceeds 25 mM, the particle size decreases (28). In another study (29), a self-assembled nanovaccine based on synthetic peptide conjugation of IKVAV-PA and OVA shows that different concentrations have an effect on vaccine characteristics including size, zeta potential, and encapsulation efficacy. Therefore, optimization of the formulation that is used for chemical reaction is a critical step for producing the best-performing SANP.

To summarize, one of the important functions of SANPs as tumor antigen carriers is to effectively deliver tumor antigens to LNs and then induce durable and effective specific-tumor immunity and immune memory. Aside from the fact that the encapsulation process protects antigens from the effect of phagocytosis or protease degradation, facilitates longer circulation time, and can generate effective immune responses (30–32), modularization with surface-attached antigens often elicit superior immune responses in comparison to encapsulated antigens perhaps owing to intracellular processing (33). Thus, composition, size, shape, and surface characteristics are key factors in ensuring stable delivery and inducing an immune response. Better biocompatible materials need to be selected to prevent their rapid removal. Particle size, as well as surface area (related to the load of the antigen), plays a critical role in DC phagocytosis and immune induction, which can be determined by controlling reaction conditions. During the design, attention should be paid to the control of chemical reaction conditions, while avoiding the infiltration of impurities, which may cause toxicity or damage the stability of the structure.

## 3 Several SANPs for vaccine design

We can classify SANPs for vaccine design from multiple perspectives. From a general point of view, we can classify them according to the method of self-assembly: dynamic self-assembly and static self-assembly (15). According to the constituent elements, SANPs can be divided into protein-based biological entities and non-protein-derived synthetic particles (23). In this review, we briefly introduce several vaccine designs based on the different components of SANPs (**Figures 1A, B**; **Table 1**).

**Table 1:** Summary of SANPs that have been explored as delivery platforms for vaccines.

NP type	Pathogen/disease	Antigen/adjuvant	Animal model	Reference
**Polymer**	Cancer	OVA	BALB/c mice	(34)
	HPV-related cancers	human papillomavirus (HPV) E7 protein-derived peptide	Mice	(35)
	Cancer	Ovalbumin	Female C57BL/6 mice	(36)
	Cancer	MUC1	Mice	(37)
	Cancer	R837	BALB/c mice	(38)
	Cancer	TLR7/8	C57BL/6 mice	(39)
VLPs	Breast cancer (BC)	Aberrantly glycosylated mucin-1	Mice	(40)
	Breast cancer	HER2 ECO	Mice	(41)
	Breast cancer	IGF-1R	Mice	(42)
	Cancer	Influenza virus, hemagglutinin, neuraminidase	BALB/c mice	(36)
	Cancer	HPV16 E6	C57BL/6 mice	(43)
	Pancreatic cancer	hMSLN	C57BL/6 mice	(44)
	Pancreatic cancer	mMSLN	C57BL/6 mice	(45)
	Melanoma	Gp100	C57BL/6 mice	(46)
	Melanoma	LCMV-TT830-843	C57BL/6 mice	(47)
Self-assembled peptides	Cancer	Antigen peptide SL8 (SIIN-FEKL), O antigen polysaccharides (OPS)	BALB/c mice, Cynomolgus monkeys	(36)
	HER2+ cancer	HER2	Mice	(48)
	Cervical cancer	HPV-L2	Mice	(49)
	Glioblastoma	EGFRvIII, PADRE, OVA	Mice	(50)
Liposomes	Cancer	—	Murine melanoma model	(51)
	Cancer	HER-2/neu-P5	BALB/c mice	(52)
	Cancer	Mycolic acid	C57BL/6 mice	(53)
	Pancreatic tumor	CpG-DNA	C57BL/6 mice	(54)
	Melanoma	OVA RNA	C57BL/6	(55)

### 3.1 Polymer-based SANPs

Polymer NPs are one of the earliest NPs used as drug delivery systems. They are often used as a material for packaging drugs like nanocapsules and nanospheres because they are non-toxic and have good biocompatibility. They can also be biodegraded into harmless alcohols and other low-molecular-weight products (21, 56). Polymers that have been developed for drug delivery can be classified into synthetic polymers and natural polymers. Common synthetic polymer NPs include polylactic acid (PLA), chitosan, polylactide glycolide acid (PLGA), polyglutamic acid (PGA), and polyethylene glycol (PEG), which are all typical polymers (11). Natural polymers such as albumin have been used to carry paclitaxel and can be used to treat metastatic cancer in the clinic (21, 57). The variety and adaptability of polymer carriers increase their therapeutic efficacy by controlling drug release, long cycle characteristics, tissue or cell targeting, and stimuli responsiveness (58).

Chemical modification of the polymer system helps to prolong the retention time in the blood, reduce widespread distribution, and target tissue or specific cell surface antigens with targeted ligands (peptides, aptamers, antibodies/antibody fragments, and small molecules). NPs based on PLGA are currently one of the most widely studied adjuvants for the effective delivery of antigens, and PLGA is a biodegradable copolymer composed of lactic acid and glycolic acid linked through ester bonds (58). DSPE-PEG2000-Gal was synthesized in an NHS-ester crosslinking reaction, which can be modified to the surface of PLGA to combine the advantages of biodegradable polymeric NPs and biomimetic liposomes, by using a water-in-oil-in-water (W/O/W) double-emulsion solvent evaporation method. PLGA polyester can be degraded from certain substances *in vivo* into lactic acid and glycolic acid so that the internal immunogen can be released continuously; in this way, cells are stimulated multiple times to avoid the need for multiple vaccinations (59, 60). For example, Po et al. designed OVA antigen-loaded PLGA NPs, glycosylated poly(lactic-co-glycolic acid) NPs loaded with the SIINFEKL peptide (OVA) as a tumor-specific antigen, and CpG oligodeoxynucleotide (CpG) as an adjuvant for an effective DC-targeted cancer vaccine (61). The biodistribution and antitumor efficiency of the SANP vaccine had a longer retention time compared with other groups. Geoffrey and his colleagues linked TLR-7/8a to HPMA-based polymers particles (Poly-7/8a) with high agonist density (8–10 mol% TLR-7/8a) and induced substantially higher LN cytokine production [which led to a ~400-fold higher level of TLR-7/8a (AUC) in draining LNs] compared with unformulated small-molecule TLR-7/8a (SM 7/8a) (62).

### 3.2 Lipid-based SANPs

The versatility and plasticity of a liposome-based delivery system are the main reasons why liposomes can act as vehicles to directly deliver drugs to cancer cells (63, 64). Liposomes are natural or artificial biodegradable phospholipid bilayer vesicles surrounded by an amphiphilic lipid bilayer with an internal water core (14, 65). It has been proven that liposomes have a good lifespan in the blood, allowing them to accumulate in lesions where blood vessels are damaged (65). This is conducive to the specific targeting of the antigen or cargo attached to the surface of the carrier to the disease site, resulting in a powerful therapeutic effect. In vaccine designs, antigenic peptide determinants can be encapsulated in lipid bilayers, and the immunogenic peptides can be protected by lipid bilayers and not degraded by enzymes (60). In addition to being a delivery agent, it also acts as a potent immune adjuvant to induce the innate immunity of the immune system (66).

One of the most classic cationic liposomes is 1,2-dioleoyl-3-trimethylammonium-propane (DOTAP) (67). It is a cationic monounsaturated phospholipid that has been found to activate DCs, thereby promoting endocytosis and antigen delivery in APCs, leading to MAP kinase, ERK activation, and induction of chemokines (68). Reactive oxygen species (ROS) were induced by cationic DOTAP liposomes from mouse bone marrow DCs (BMDCs), and ROS were involved in the expression of DOTAP-induced costimulatory molecule CD86/CD80, indicating the antitumor activity of ROS for vaccine induction (69). Recently, cationic liposomes have been shown by several studies to be promising vaccine delivery platforms for therapeutic cancer vaccines because of their ability to increase the immunogenicity of antigen-based vaccines (70–72). The liposome-based synthetic long peptide (SLP) vaccine has been shown to efficiently induce functional antigen-specific CD8^+^ and CD4^+^ T cells and to effectively eliminate tumor in two different tumor-bearing mouse models (71, 72). Heuts et al. investigated cationic liposomes loaded with 15 SIINFEKL T-cell epitope-containing SLPs; cationic liposomes efficiently delivered the SLPs to DCs that subsequently activated SIINFEKL-specific CD8^+^ T cells, indicating the improved immunological activity of the SLPs (73). In addition to vaccines based on peptide antigens, liposomes have also been used to deliver RNA-based vaccines (74). Jinjin et al. developed an LN targeting lipid (113-O12B), which was used to deliver an OVA-encoding mRNA cancer vaccine against a melanoma mouse model. In addition to exhibiting better expression in LNs compared with ALC-0315 (a synthetic lipid used in the COVID-19 vaccine Comirnaty), the vaccine also promoted mRNA expression in APCs, but the potential hepatotoxicity needs to be verified (75). Matthias et al. found that incorporation of different adjuvants (LPS) will improve the potency of the lipid-based SANP mRNA vaccine, resulting in B16 F10 melanoma tumor shrinkage and extended survival of the tumor-bearing mouse (76).

### 3.3 Peptides-based SANPs

Most of the vaccines based on biological materials have been extensively studied. Peptide-based vaccines can cause a more concentrated immune response over protein sequences or inactivated viruses (77). The primary structure and site modification of peptide nanomaterials are based on amino acid design (78), which can be designed into self-assembled peptide nanostructures with unique secondary structures (79). The distribution of charges around amino acids will affect the performance of the nanoscaffold and the speed of self-assembly (80, 81). Compared to other types of vaccine platforms, self-assembled peptides are less toxic than polymer-based SANPs (82), and they are generally considered to have higher stability than lipid-based SANPs (83). The self-assembled supramolecular structures can be perfect modules for constructing nanofibers, nanotubes, nanocapsules, and nanomicelles (84). Self-assembled peptides can be coupled with peptide epitopes without affecting their respective characteristics; finally, immunogenic, biocompatible, stable, and self-adjuvanted vaccines can be designed. In recent years, due to their unique chemical, physical, and biological properties, self-assembled peptides have received increasing attention from scientists. Geoffrey et al. developed a vaccine platform (SNP-7/8a) that is chemically programmed to self-assemble into homogeneously sized (~20 nm) NPs, based on charge-modified peptide-TLR-7/8a conjugates (85), which targets patient-specific neoantigens’ physicochemical variability and presents challenges in manufacturing personalized optimal cancer vaccines to induce anticancer T cells. This approach promotes T-cell immunity by promoting the uptake and activation of APCs through the precise loading of multiple peptide neoantigens linked to TLR-7/8a (adjuvant) in NPs. Wei et al. developed a self-assembled peptide system as a novel adjuvant that can efficiently deliver two antigens (MAGE/NY-ESO-1) (86). The SANPs not only widened the response range within the same molecule but also significantly prolonged the plasma half-life of single antigenic peptides, which can be a broad-spectrum candidate for effective breast cancer therapy.

### 3.4 Virus-like particles and SANPs

Similar to self-assembling peptides, virus-like particles (VLPs) are a complex structure composed of viral structural proteins that have the ability to self-assemble when they are recombined and expressed (87, 88). The main difference between VLPs and native viruses is that they lack viral genetic material, so they cannot replicate or induce infection (89). Therefore, VLPs are one of the safest candidates for attenuated or inactivated pathogen-based vaccines. VLP epitopes have repetitiveness and high density, which is the main reason why they can cause a strong immune response (90). This also shows that it has the potential for effective recognition, cell absorption, and processing of the host immune system (91). Its granularity further consolidates this advantage, and its proper size (less than 40 nm) is particularly suitable for obtaining DCs (92).

In the past 10 years, several VLP-based preventive vaccines have been approved for marketing, including human papillomavirus (HPV) and hepatitis B virus (HBV). VLPs based on HPV are formed by a single structural protein, and there are also more complex VLPs, such as the VLPs of the Reoviridae family composed of two to four different proteins (93, 94). When selecting a VLP production platform, it is necessary to fully consider the modification requirements of the protein and select an appropriate expression system. Common production platforms include *Escherichia coli*, yeast, insect cells, and mammalian cells (87). Consider safety or production times and costs, plant-derived viruses such as cowpea mosaic virus (CPMV), tobacco mosaic virus, and potato virus X (PVX) were successfully used as a carrier/platform to present foreign epitopes (95, 96). Sourabh et al. developed CPMV and PVX as vaccine platforms against HER2^+^ malignancies (48). CPMV generated a stronger selective induction of cytokines and chemokines than empty CPMV, a VLP composed of CPMV capsid without nucleic acids, in naïve mouse splenocytes (95). Valeria et al. evaluated the function of bacteriophage MS2 VLP, which was used to display an extracellular loop of xCT protein. In a metastatic breast cancer model, the VLP-based vaccine reduces tumor metastasis by eliciting a strong antibody response and is well-tolerated (97).

### 3.5 Other SANPs

Polymeric lipid hybrid nanoparticles (PLHNs) are an emerging carrier platform, which can overcome the limitations of liposomes and polymeric NPs. The biodegradable polymeric matrix core has a larger surface area and more stability than liposomes, as well as a controlled release, with the lipid layer surrounding the polymeric core being a highly biocompatible shell (98). Although this self-assembly method can obtain an optimal conformation, the gap between synthetic immunotherapy and endogenous immunity has been the reason for imprecise and inefficient treatment. The strategy of using natural biomembranes to camouflage NPs to mimic the properties and functions of biological interfaces has been widely explored. For example, Chen and colleagues developed a cancer vaccine based on PLGA and a hybrid membrane, which was from the fusion of *E. coli* cytoplasmic membranes and an autologous tumor membrane (99). The hybrid membrane NPs can induce DC maturation, eliciting strong tumor-specific immune responses. Wang et al. developed a bacterial outer-membrane vesicle–cancer cell hybrid membrane and successfully tested it on HPDA NPs. The immune activation properties derived from the source membrane can rapidly stimulate DC maturation in LNs (100). Existing self-assembled nanovaccines that can be biosynthesized *in vivo* have been investigated. Pan and colleagues developed a Nano-B5 platform that can be synthesized *in vivo* and is a biosynthetic self-assembled nanovaccine based on a whole protein (101). They used genetic engineering technology to express a fusion module monomer in the periplasmic space of *E. coli* DH5α cells, in which the protein could be folded correctly. The monomer consists of B5 subunits of cholera toxin (CTB) as an adjuvant internal module, and a C-terminal trimer peptide is linked through a connecting peptide (GGSG). The antigen part is made up of O antigen polysaccharides (OPS) formed by linking the pentameric sugar unit oligosaccharide substrate produced by the cell. They can self-assemble into NPs with a size of approximately 25–50 nm in the cytoplasm without affecting their unique properties.

## 4 The self-adjuvanticity of SANPs

The regulatory mechanism of adjuvants is related to the direct or indirect stimulation of APCs (60). The maturation of DCs stimulated by adjuvants and the immune response to host antigens are two major prerequisites for therapeutic cancer vaccines to work (102) (**Figure 1D**). DCs are an important messenger between CD4+ T helper cells and CD8+ T cells, and are associated with triggering further adaptive immune responses (103). The mechanistic routes of adjuvants were summarized by Schijns (1): adjuvants facilitate antigen uptake, presence, and transport by antigen-capturing and -processing cells; (2) adjuvants prolong the time of antigen storage and release; (3) adjuvants activate innate immune cells to release cytokines through targeting the pattern recognition receptor (PRR); and (4) adjuvants mimic danger signals to stimulate the APCs (104). Toll-like receptors (TLRs) are a popular target for adjuvant research, because of their involvement in identifying pathogen-associated molecular patterns (PAMPs) (60). In the process of the adaptive immune response, adjuvants can fully stimulate CD4+ T cells, but the initiation of CD8+ T cells requires more complex immunological activities (60).

Conventional adjuvants like aluminum salt have a certain effect on inducing T helper 2 (Th2) cell responses, not Th1 or cytotoxic T lymphocytes. Therefore, it is assumed that this type of adjuvant would fail to activate the immune response required to cause tumor killing (105). Live-attenuated viral vaccines generally do not need an adjuvant because they can mimic a natural infection. Therefore, virus-like NPs can be regarded as an exogenous antigen to be presented by MHC class II molecules, which can also combine with MHC class I molecules by cross-presentation to activate humoral and cellular immunity (106). In general, self-adjuvanticity of SANPs relies on the formulation characteristics of the particulate’s structure. Because SANPs have a bio-particulate structure that is usually taken up efficiently by DCs, these SANPs with a perfect phenotype are often preferred as adjuvants of cancer vaccines and have been applied in multiple immunotherapy programs involving vaccines. A variety of SANPs have been reported to induce powerful antigen-specific T-cell responses through targeting TLRs. Yoshikawa et al. demonstrated that poly-γ-glutamic acid could activate APCs and induce a potent antigen-specific T-cell response through the TLR4 and MyD88-dependent signaling pathway (107). Mesa et al. found that TLR4 on DCs can be activated by small-sized proteoliposomes (VSSP), which are produced by the hydrophobic interaction of GM3 ganglioside with the meningococcal outer-membrane protein complex (108). Additionally, Luo et al. developed heterocyclic lipid SANPs with self-adjuvanticity that could activate type I interferon-stimulated genes by inducing STING activation but not the TLR or MAVS pathway (109). The potent T-cell activation with checkpoint inhibition showed great synergy with 100% survival over 60 days in a TC-1 tumor model. Furthermore, maintaining safety and reducing the vaccine dose are important factors for cancer patients who can benefit from the advancement of SANP technology.

Notably, the potential of SANPs can act both as delivery systems for vaccine antigens and as immunomodulators as previously reported (110). Several *in situ* vaccine (ISV) approaches have been suggested, with vaccine regimens including SANPs, Flt3L, and TLR agonists (111–113). However, the development of nanomedicine has fully demonstrated the advantages of SANP with both physiological activity and physical properties. A semiconducting polymer nano-immunomodulator (SPNI) has been previously reported (114), which is self-assembled by a polymer conjugated with a TLR7 agonist *via* an acid-labile linker. The semiconducting polymer contributed to tumor eradication and immunogenic cancer cell death through a near-infrared (NIR) absorbing to exert photodynamic effects. This way, the synergistic action of released immunogenic factors and acidic TME-activated TLR7 agonist can not only kill tumor cells directly but also serve as an *in situ* generated cancer vaccine to evoke strong antitumor activities. This SANP-based design refreshes the acquired route and presentation of traditional vaccines and improved therapeutic cancer vaccines.

## 5 Self-assembled nanoparticle platforms are a promising strategy in future cancer therapy

Reports in recent years have shown us a variety of strategies that try to improve the immunogenicity of vaccines by using the powerful immune stimulation of SANPs in the tumor microenvironment. However, the great obstacles to the production of the specific killing effect of cancer vaccines include the genomic and phenotypic heterogeneity of cancer and the immunosuppressive effect of the tumor microenvironment. Furthermore, intertumoral and intratumoral heterogeneity provided a basis for therapeutic resistance (115). The advent of personalized therapeutic cancer vaccines provides a strategy by targeting personal tumor antigens (neoantigen), and the success of the neoantigen platform was achieved by tumor mutational burden (TMB), which is the total number of mutations per coding area of a tumor genome (116, 117). Specifically, the clinical significance from immune checkpoint inhibitor therapy has also been shown to correlate with the higher TMB, within several tumors including NSCLC, small cell lung cancer, melanoma, and colorectal cancer (118–123). It is reasonable to assume that high TMB induces high densities of neoantigen-specific tumor-infiltrating lymphocytes, leading to tumor cell secretion of IFN-γ and upregulation of PD-L1, while their relationship across the entire human cancer spectrum remains unclear (124, 125). If the combination of the SANP platform and a neoantigen is feasible, then it will bring unexpected results. However, considerable challenges to this approach include the cost and time required to achieving this high degree of personalization.

### 5.1 SANPs combined with other treatments

In established cancers, therapeutic vaccines will require co-treatments to overcome immune evasion and to become fully effective. However, in clinical practical applications, these drugs have failed to eliminate immunosuppressive cells (126–130) and may have inadvertently created immune evasion of tumor cells in the tumor microenvironment (131–134), resulting in suboptimal therapeutic cancer vaccine efficacy. Nanotechnology interventions have overcome the limitations of current conventional chemotherapy, including poor biological distribution, cancer cell resistance, and severe systemic side effects, dramatically changing the treatment of cancer. The properties of these delivery systems have been adjusted to enhance the stability of delivery to tumors; for example, the hydrophilicity of NP surfaces provides longer cycle times through stealth, positively charged surfaces that can enhance the internalization of cancer cells. Delivery systems based on SANPs are commonly used to deliver anticancer drugs, gene drugs, targeted drugs, and stimulus-responsive drugs (26). Peptide self-assembled fibers are popular materials for anti-cancer drug delivery systems. Compared with traditional cancer treatment, peptide hydrogels can slowly and directly release chemotherapeutic drugs to cancer tissues. For example, Li and colleagues successfully developed NPs through the self-assembly of hyaluronic acid (HA)–cystamine–cholesterol (HSC) conjugates, in which IR780 was simultaneously incorporated (HSCI NPs) (135). After cellular uptake, HSCI NPs are broken down by the reaction of cystamine with overexpressed GSH. The released IR780 will induce a fluorescent “on” transition, which can be used to effectively image the tumor site. After irradiation with an 808-nm laser, programmed photoactive therapy (PPAT) can be realized, and the ROS generated therein will generate photodynamic therapy (PDT).

One of the main advantages of NP-based delivery systems is that multiple drugs with similar or synergistic effects can be encapsulated in a single nanoformulation (136). However, NPs that enter the systemic circulation by intravenous injection usually absorb the majority of macrophages through the mechanism of phagocytosis (137). To avoid similar recognition, the stealth substance PEG can be wrapped in the outer layer of the NP to improve the efficiency of drug delivery to target cells and tissues (138). However, people are worried about the production of PEG antibodies. Of course, this can be solved by using other substances instead of PEG, such as zwitterionic polymers (139). Akhilesh et al. synthesized PGS and PEG copolymer elliptical NP PGS-co-PEG by chemical synthesis based on the elastic properties and biocompatibility of polyglycerol sebacate (PGS) (13). Subsequently, bovine serum albumin was used as a model protein to encapsulate and self-assemble using the nanoprecipitation method. The encapsulation rates of PGS-20PEG and PGS-40PEG were 88.5% and 91%, respectively. As the outer surface of NPs, PEG provides good stability and stealth properties, which play an important role in improving pharmacokinetics, drug delivery efficiency, and drug targeting.

### 5.2 The prospect of combination immunotherapy

#### 5.2.1 PD-1 blockers

The common goal of cancer vaccines and other immunotherapies is to stimulate effective antigen-specific immunity. The combined use of cancer vaccines and other immunotherapies also has a large experimental and theoretical basis. Immune checkpoint inhibitors (ICIs) such as PD-1 blockers have been shown to have exceptional effectiveness against solid tumors by preventing PD-1/PD-L1 binding; however, the monotherapy approach is not sufficient and most patients either do not respond or eventually relapse. ICIs have emerged as a breakthrough approach in cancer therapy due to the exposure of the tumor microenvironment to immunosuppression and provided a strong rationale for the combination with vaccines.

For advanced tumors, they are generally more resistant to ICIs and vaccination alone has been ineffective for invasive cancer. A randomized clinical trial has demonstrated the safety and immunogenicity of this treatment regimen, and the contribution of vaccination to the tumoricidal effects of PD-1 inhibition has also been confirmed, including that for advanced melanoma, non-small cell lung cancer, or bladder cancer (140). Recently, a therapeutic HPV-16 SLP vaccine combined with the PD1 inhibitor in patients with HPV-16-positive cancer was shown with only apparent additive effects from each agent without increased immune adverse events, relative to PD-1 monotherapy (141). Based on the discovery of the synergy of the two treatment modalities, we can combine two routes of administration into one treatment system or fuse them into a single expression system. Le et al. modified an anti-PDL1 antibody to NP surfaces, which involve adjuvant-loaded NPs that were prepared by entrapping imiquimod (IQ) in photoresponsive polydopamine nanoparticles (IQ/PNs) (142). Following NIR irradiation, mice treated with PDL1Ab-IQ/PNs not only resulted in primary tumor ablation, but also completely prevented secondary tumor growth at distant sites, with 100% survival for up to 150 days. Recently, researchers from New York have modified chimeric antigen receptor T cells (CAR-T) to secrete PD-1 blocked single-chain variable fragments (scFv); these scFv-secreting CAR-T cells play paracrine and autocrine roles to improve the antitumor activity of CAR-T cells and bystander tumor-specific T cells in syngeneic and xenogeneic mouse models of clinically relevant PD-L1^+^ hematological and solid tumors (143). These examples illustrate the complexity of tumor resistance to vaccines and immunotherapies, and highlight how multiple modalities will be required for therapeutic vaccines or other immunomodulatory therapies to overcome suppressive TME.

#### 5.2.2 Chimeric antigen receptor T-cell therapy

Directly inducing the immune response of tumor lesions or a targeted effector T-cell response can be achieved through vaccination approaches combined with adoptive cell therapy. Due to the remarkable success of CAR-T cell therapy in hematological malignancies but not in solid tumors, the combination with nanotechnology may be more attractive (144, 145). Conventional NPs can boost CAR-T therapy by emerging as carriers for CAR-T to enhance targeting, or as a tool to enhance transfection efficiencies of CAR gene. However, SANPs can provide more potential strategies that could be used to modify CAR-T cells or as a booster vaccine to overcome the existing challenges in solid tumors. Natnaree et al. reported an approach in which cross-linked multilamellar vesicles (cMLV) could be covalently attached to CAR-T cells to deliver the A2aR-specific small-molecule antagonist SCH-58261 and the cMLVs NPs without affecting the effector and viability of CAR-T cells (146). In addition, Chenjun et al. previously established self-assembled multivalent CAR-like aptamer NPs, which can activate T cells while targeting B16 mouse melanoma tumor cells (147). The predictable result is the increase in durability of tumors and even the increase in efficacy of CAR-T against solid tumors. Towards this end, the combination of therapeutic CAR-T cell therapy with vaccines, such as DC vaccine, RNA vaccines, or novel approaches, was tentatively developed. The main argument of this treatment plan is synergy after immune activation. Reinhard et al. developed a CAR-T cell-amplifying RNA vaccine with lipid-based SANPs (148), by inducing DC natively displayed CLDN6, a tetraspanin membrane protein that is involved in tight junction formation (149), to promote cognate and selective expansion of CLDN6-CAR-T cells. However, this vaccine did not directly participate in the antitumor immune response in this process. Additionally, co-delivering CAR-specific ligands that boost CAR-T cell numbers and functionality *in vivo* with low toxicity decorate the APCs in the LN and provide critical priming signals to the CAR T cells (150). In general, it is necessary to pay attention to the crucial problems and obstacles of clinical progress, concentrate on resources and promote the understanding of the inconclusive interactions of the immune system, and develop optimal treatment schemes for cancer patients.

## 6 The challenge of SANPs in cancer vaccine

Today, most of the existing production processes of self-assembled nanovaccines are synthetic methods or semibiological technologies based on certain specific cells (101). The potential advantages of these strategies include reduced production time and cost. However, chemical synthesis and semibiosynthesis will limit the preventive and therapeutic effects of vaccines, which must face the difficulty of displaying versatile antigens on these proteinaceous NPs, especially glycan antigens with complex structures. Of course, the toxicity of individual materials is still present. A series of local events are generally considered as tissue or cell response continuum, such as injury by injection or implantation, acute inflammation, chronic inflammation, formation of granulation tissue, severe cytotoxicity, foreign body reaction, and fibrosis following implantation of microspheres (59, 151, 152). The method of using protein-based fully biosynthetic vaccines is still being explored, and this strategy still faces many difficulties related to antigen display. The preparation process of some SANPs used for antigen and drug delivery involves a series of chemical reactions; interestingly, the molecular interaction between the drug and the carrier does not break its inherent activity with the self-assembly process (17, 153, 154), which is conducive to the carrier and cargo fully playing their respective roles *in vivo*.

## 7 Conclusion

Future research on therapeutic cancer vaccines will focus not only on individualization of antigen for every tumor, but even more on the proper vaccine platform to maximize their impact. Breakthroughs in antigen delivery platforms have increased our capacity to personalize vaccines. Several supramolecular assembled programmable nanomedicines reported in recent years to improve immunotherapy efficiency and the emerging technology have made it possible to dissect the TME in-depth (113, 155, 156). To further inform the design of future treatment platforms, mechanistic analyses can be collected from previously failed and some clinically significant vaccine trials. Many ongoing studies using a range of different vaccine delivery platforms and combination therapies to alleviate tumor resistance have the ultimate goal of inducing effective, long-lasting, tumor-specific immunity in cancer patients.

SANPs composed of each material have specific advantages. For example, polymers have good stability and excellent biocompatibility, and peptide NPs are expected to achieve comprehensive biosynthesis in the future. The high loading, low toxicity, and high biocompatibility of most SANPs make them more valuable in vaccine development than traditional adjuvant vaccines. At the same time, vaccines based on various NPs also have shown good results in animal models, which provides a huge impetus for the development of many vaccines against complex diseases in the future. Although many satisfactory experimental results have been produced in the field of self-assembled nanovaccines, the current understanding of the physical and chemical characteristics of nanomaterials and their interaction with physiological systems is limited, and the optimal synthesis, synthesis of nanomaterials, and chemical modification are unclear (157). To provide better protection, a self-assembled nanovaccine must be able to induce a series of immune responses. Although existing functionalized NPs can be internalized by immune cells, the effect of the sites of many functional proteins for the uptake of APCs is still unknown (158). It should be recognized that the idea of carrying antigens on the surface of SANPs to induce immune responses proves to be promising in the least. In the future, we are expected to load more antigens on the surface of multivalent NPs to drive specific and strong CD4+ T-cell and/or CD8+ T-cell responses. If an NP platform can resolve distribution barriers in the future, the age of enlightenment of nanotherapeutics is nearing its end.

## Author contributions

TS and FM conceived the structure of this review. TS wrote and edited the manuscript. MS collected and prepared the related figures. FM and CL reviewed and made revisions to the manuscript. All authors contributed to the article and approved the submitted version.
